# Application of Pneumatic Lithotripter and Holmium Laser in the Treatment of Ureteral Stones and Kidney Stones in Children

**DOI:** 10.1155/2017/2505034

**Published:** 2017-02-19

**Authors:** Marcin Życzkowski, Rafał Bogacki, Krzysztof Nowakowski, Bartosz Muskała, Paweł Rajwa, Piotr Bryniarski, Andrzej Paradysz

**Affiliations:** Department of Urology, School of Medicine with the Division of Dentistry in Zabrze, Medical University of Silesia, Katowice, Poland

## Abstract

*Objective.* Treatment options for urolithiasis in children include URSL and RIRS. Various types of energy are used in the disintegration of deposits in these procedures. We decided to evaluate the usefulness of URSL and RIRS techniques and compare the effectiveness of pneumatic lithotripters and holmium lasers in the child population based on our experience.* Materials and Methods.* One hundred eight (108) children who underwent URSL and RIRS procedures were enrolled in the study and divided into two (2) groups according to the type of energy used: pneumatic lithotripter versus holmium laser. We evaluated the procedures' duration and effectiveness according to the stone-free rate (SFR) directly after the procedure and after fourteen (14) days and the rate of complications.* Results.* The mean operative time was shorter in the holmium laser group. A higher SFR was observed in the holmium laser but it was not statistically significant in the URSL and RIRS procedures. The rate of complications was similar in both groups.* Conclusions.* The URSL and RIRS procedures are highly efficient and safe methods. The use of a holmium laser reduces the duration of the procedure and increases its effectiveness in comparison with the use of a pneumatic lithotripter.

## 1. Introduction

Treatment options for urolithiasis in children include open and laparoscopic procedures, extracorporeal shock wave lithotripsy (ESWL), percutaneous nephrolitholapaxy (PCNL), and ureteroscopic lithotripsy (URSL) for ureterolithiasis and retrograde intrarenal surgery (RIRS) when deposits are located within the pelvicalyceal system. Lately, the indications for ureterorenoscopic treatment have included mainly acute states (pain on the type of renal colic, obstruction of the upper urinary tract), lack of progress after ESWL, and states in which the disease led to renal failure requiring renal replacement therapy [1, 2]. Also, the age of the patients was a limitation, making this method a tool for treating urolithiasis in older children. A very high efficiency of ureterorenoscopic lithotripsy in the treatment of urolithiasis in the adult population stimulated attempts of using this technique wider in pediatric urology [3–6]. The technological advance, including the miniaturization of endoscopic equipment and the introduction of holmium laser, as well as urologists' growing experience allowed not only for reducing the lower age limit for children undergoing treatment but also for improving the effectiveness of the disintegration of stone deposits located in the upper urinary tract.

## 2. Aim of the Study

1. Comparative analysis of the effectiveness of the pneumatic lithotripter and the holmium laser in the disintegration of stones in the upper urinary tract in children.
2. Evaluation of the effectiveness and safety of URSL and RIRS endoscopic techniques in the treatment of urolithiasis in children.

## 3. Materials and Methods

We conducted a retrospective analysis of the results of ureterorenoscopic lithotripsy performed in 108 children aged 3 to 17 during the period of January 1995 to December 2015 due to radiopaque stones in the upper urinary tract. The follow-up period ranged from 3 to 240 months.

The patients were divided into two groups: the first consisted of children operated on with the use of a pneumatic lithotripter (group I) and the second comprised children treated using a holmium laser (group II). Pneumatic lithotripsy was performed in 65 children (60.19%)—36 girls and 29 boys, including 46 URSL and 19 RIRS. From February 2009 until December 2015, 43 children (39.81%) were operated on using a holmium laser—27 girls and 16 boys, out of which in 17 cases the deposits were located in the ureter—URSL procedure, and 26 in the pelvicalyceal system, the RIRS procedure. The procedures were carried out by two experienced pediatric urologists. Before surgery, all patients underwent routine laboratory tests: blood count, electrolytes, serum creatinine, urinalysis, and urine culture. Diagnostic imaging involved abdominal ultrasound, abdominal X-ray (KUB), and intravenous pyelography (IVP) or noncontrast enhanced computer tomography (CT) of the abdomen and pelvis. Voiding cystourethrography was performed in the preoperative period in 24 children (36.92%) in group I and 13 children (30.23%) in group II with recurrent urinary tract infections in the medical history. The diameter of the urinary stones was between 5 mm and 20 mm; the stone surface areas (SA) of the deposits in all groups are shown in Tables 2 and 3. In one case, there was a simultaneous bilateral ureterolithiasis and bilateral staghorn nephrolithiasis. Urinary tract defects were found in 10 patients in group I (15.38%) and in 9 children in group II (20.93%) (Table 1).

In patients in the analyzed groups, deposits were located within the ureter and the pelvicalyceal system—in the renal pelvis and in the group of upper and middle calyces. Procedures were performed using a 4,5 F, 6,5 F, and 9,5 F ureterorenoscope. In order to disintegrate the deposits, the Swiss Lithoclast EMS pneumatic lithotripter and, since 2009, a Sphinx Holmium-Yag laser with 18 W power, 12 Hz frequency, and 1.5 J energy and a 200 *μ*m fiber were used. The procedures were performed in the lithotomy position, using the video track. All patients were operated on under general anesthesia. Before the surgery, antibiotic prophylaxis was used. The ureterorenoscope was carefully introduced into the bladder under visual guidance and the ureteral orifice was located. The subsequent phases of the procedure were performed under fluoroscopic guidance. The guiding wire was introduced into the ureteral orifice, followed by the ureterorenoscope. The access sheath was not utilized in order to avoid enlarging the ureteral orifice. During the procedure, the ureterorenoscope was moved carefully within the lumen of the ureter using visual and fluoroscopic guidance. The disintegration of calculi deposits was performed with the use of a pneumatic lithotripter or a holmium laser lithotripter. The laser parameters were adjusted based on the degree of the deposits' hardness. The remaining deposits with a diameter up to 2 mm were left behind to be expelled spontaneously, and those over 2 mm were removed using endoscopic pliers or a Dormia basket. Self-adhesive “double J” ureteric catheter was left in the ureter in cases of a mechanical or thermal damage to the ureter mucosa, its swelling at a deposit site, or swelling of the ureteral orifice. Postoperative follow-up in case of remaining small deposits (up to 2 mm) included hydration, antispasmodic medication (papaverine hydrochloride i.v., drotaverine hydrochloride p.o.), analgesics (acetaminophen, ibuprofen), and rapid mobilization of the child.

The procedure time was assessed for both groups. The effectiveness of the procedure was assessed based on the percentage of patients in each group with a complete disintegration of the calculi deposit, referred to as stone-free rate (SFR), which is defined as a complete disintegration of the calculi with potential remaining deposits with a diameter up to 2 mm up to 14 days after procedure. The assessment of the efficacy was based on imaging studies performed in the postoperative period. On day 1 after the operation, a urinary ultrasound and a plain abdominal X-ray (KUB) were performed. After 10–14 days, a urinalysis, urine culture, serum creatinine level, and ultrasound of the urinary tract were performed in an outpatient clinic. Urinalysis and urinary ultrasound were subsequently performed at 3 and 6 months after the procedure and then once a year. Decisions to undertake other investigations were made on an individual basis, depending on the patient's clinical status.

The indications to perform voiding cystourethrography included persistent bacteriuria, dilatation of the pelvicalyceal system, and the proximal or distal part of the ureter observed in the ultrasound examination during the postoperative period.

Complications were classified as early or late, depending on the time of onset. Early complications (up to 14 days after procedure) included the following: fever, pain of the renal colic type, microscopic or macroscopic hematuria, and urinary tract infection. Late complications involved a de novo diagnosis of vesicoureteral reflux and ureteral strictures.

All data were checked for normality with the Kolmogorov-Smirnov test. Data distribution was not normal. In order to analyze the continuous variables without normal distribution, the Mann–Whitney nonparametric *U*-test was used. For the analysis of the categorical variables, chi-square tests were used. The statistical examination was conducted with Statistica StatSoft version 9.0. *p* values lower then 5% were considered as statistically significant.

## 4. Results

The mean operative time was, respectively, 56 minutes for the use of a pneumatic lithotripter and 34 minutes for the use of a holmium laser (*p*  –  0.04) (Table 4). The complete disintegration of the deposits (stone-free rate—SFR) in the ureter was obtained in 40 cases (86.96%) in group I and for 17 patients (100%) in group II. Among the remainder of the patients in group I, in 1 case (2.17%), partial disintegration and extraction of the debris with grasper were performed, and in 3 children (6,52%) tiny shards for spontaneous expulsion were left. In 2 children (4.35%), the treatment failed (Table 5). In the case of kidney stones, complete disintegration (SFR) was obtained in 9 cases (47.37%) in group I and in 23 cases (88.46%) in group II. Fragmentation of the stones and debris extraction with grasper were performed in 6 cases (31.58%) in group I and in 1 patient (3.84%) in group II. Fragmentation of the plaque, leaving small debris, occurred in 4 patients (21.05%) in group I and in 2 cases (7.7%) in group II (Table 6). Appropriate postoperative treatment included the following: hydration, diastolic drugs, and painkillers allowed spontaneous expulsion of small residual debris. “Double J” catheter implantation was necessary in 7 patients in group I and in 3 cases in group II (*p*  –  0.03). The mean follow-up was 82 months (3–240 months). The following complications were reported for group I: fever in 12 children (18.5%), pain in the type of renal colic in 13 children (20%), transient hematuria in 10 children (15.38%), Clavien grade I, and urinary tract infection in 11 children (16.92%), Clavien grade II. In the case of 1 child—a 16-year-old girl with urolithiasis of the middle ureter—there was a serious complication in the form of extensive damage to the wall of the ureter during the attempt of plaque removal with the use of Dormia basket which required open surgery repair (Clavien grade IIIB). For children in group II, the following were found: fever in 9 cases (21%), renal colic in 9 (21%), transient hematuria in 5 (11.63%), Clavien grade I, and urinary tract infection in 7 cases (16.28%) (Table 7). All of these symptoms subsided after using conservative treatment. None of the patients underwent dilatation of the vesicoureteral orifice. In postoperative voiding-cystourethrography, vesicoureteral reflux was observed in 2 children after the pneumatic lithotripsy and in 1 child operated on with a holmium laser, in which the defect was not diagnosed in the preoperative tests. During a further observation, in all of the 3 children, the outflow had a spontaneous regression.

## 5. Discussion

ESWL is currently considered to be the first-line treatment for kidney stones. The limited efficacy of this technique, particularly with regard to patients with congenital urinary tract malformations, is often associated with the requirement to repeat the procedure and thus exposing the children to rehospitalizations and the risks associated with anesthesia. The Landau team noted that SFR in 3 months after ESWL was 80%, whereas 20% of patients required reoperation [7].

Endoscopic lithotripsy was initially used for urolithiasis of distal ureter and characterized by SFR efficiency of up to 97% [8]. With the development of technology and staff experience, it started to be used in the treatment of stones located in the other sections of the ureter, with the efficacy of SFR 88–100% and minimal complications [9–11].

The observations of Minevich et al. based on 71 procedures performed in children emphasised the safety of the URS procedure. There was no damage to the wall of the ureter reported, while one case of postoperative ureteral stenosis was successfully treated endoscopically [12]. Miniaturization of the equipment also contributed to reducing the need of dilatation of vesicoureteral orifice and thus the risk of developing iatrogenic vesicoureteral reflux [13, 14].

The efficacy and safety of URS in pediatric patients was confirmed by Smaldone et al. Endoscopic treatment was used in 100 children. A complete disintegration of deposits was successful in 91% of them, and only 9% required further surgery. The perforations of the ureter, observed in 4.2% of cases, were effectively provided with a temporary assumption of ureteral stent, while one case of ureteral obstruction required surgery [15].

Based on the results of our observations, we conclude that endoscopic procedures using either a pneumatic lithotripter or a holmium laser one are both safe, with a similar percentage of clinically nonsignificant complications, in both the URSL and RIRS techniques. No statistically significant differences were found between the two groups with regard to the number of complications. An apparent advantage of the laser is a statistically significant shorter procedure time in this group of patients. The one noted case of a severe complication in the form of extensive damage to the ureter wall cannot be analyzed in the context of the equipment utilized but rather with regard to the risk associated with the endoscopic technique as such.

Due to the careful introduction of the ureterorenoscope into the ureteral orifice and the choice to not utilize the access sheath in the groups of patients discussed, a low percentage of vesicoureteral reflux was found, followed by spontaneous regression.

During the postoperative period, no iatrogenic ureteral stricture was found in either group of patients, which was confirmed by IVP or contrast enhanced CT performed in patients with persistent upper urinary tract obstruction. There is a low probability of ureter stricture in patients without diagnosed obstruction.

Reports on the high efficiency of ureterorenoscopic lithotripsy began to undermine the position of ESWL as a first choice method in the treatment of urolithiasis. The ESWL has a limited effectiveness with large concretion, staghorn, cystine, and radiolucent stones [7]. The differences in favor of the URSL are particularly pronounced for deposits located in the lower ureter, due to the limitation of external generator wave penetration to the plaque owing to the presence of the pelvic bones. The advantage of URS is also shown in the context of the stone size. Nelson's team, who investigated the efficacy of ESWL, reported the SFR to be at 63% in the size of the plaque ≤10 mm, while SFR only around 25% with stone size >10 mm [16]. In their randomized trial, De Dominicis et al. randomly divided 31 children with urolithiasis of the distal ureter into URSL and ESWL groups. URSL proved to be much more effective than ESWL (94% versus 43%) [17]. The frequent failure of ESWL in children, however, is primarily associated with anatomical abnormalities of the kidney and urinary tract malformations. According to some authors, they reduce the chance of plaque removal after the first session from 64 to 31% [18, 19].

The high efficacy of the endoscopic lithotripsy for deposits located within the ureter has led to attempts to use this method in the disintegration of stones located in the pelvicalyceal system. The team of Abu Ghazaleh performed RIRS in 56 children, observing an efficacy of 94.8% [20]. Other investigators' observations confirm the high efficiency as well as the safety of this technique with SFR 88–100% [21–24].

Introducing holmium lasers to urology, as a miniaturization of equipment, has proven to be another huge step forward. Safwat et al. demonstrated the effectiveness of the laser in pediatric endourology proving up to 100% SFR for deposits located in different places of the ureter, in the absence of complications [25]. Subsequent reports proved the holmium laser with SFR 89–100% to be highly efficient [11, 22, 26, 27]. Through comparing pneumatic with laser lithotripsy based on procedures performed in 394 children, Bapat et al. showed that the percentage of disintegration is significantly higher with the use of a holmium laser (86.01% versus 97.01%), with a negligible number of events [28].

Through the utilization of laser ureterolithotripsy, we were able to achieve a complete disintegration of ureteral deposits (SFR) in 100% of the patients on the first attempt, compared with an 87% efficacy with the use of the pneumatic lithotripter. In the case of RIRS, a complete disintegration of deposits (SFR) during the first procedure was successful in 88.46% of the patients in group II and in 47.37% of the children in group I. In our opinion, particular attention should be paid to the RIRS technique utilizing a holmium laser lithotripter in cases of calculi deposits located within the pyelocalyceal system. The high efficacy of this method—reaching up to almost 90% according to our observations—as well as the absence of clinically significant complications should make it a method of choice in the case of nephrolithiasis, particularly for patients with hard deposits [29]. The advantage of endoscopic technique over ESWL, especially apparent in the SFR values achieved after the first procedure compared with ESWL, in our opinion, provides the rationale for performing the procedure under general anesthesia [30].

In the mentioned study on ureterorenoscopic lithotripsy by Smaldone et al., only 9% of the children required retreatment [15]. On the other hand, Bapat et al., comparing the use of laser lithotripsy with the use of pneumatic lithotripsy, reported that only 1.99% of the children treated with the laser required further surgery, while after the use of pneumatic lithotripter this percentage reached 13.98% [28]. In our experience, a complete deposits' disintegration or a fragmentation that reduces them to a size enabling the removal with pliers was achieved in 56 children (86.2%) in group I and 41 children (95.3%) in group II. A repeat endoscopic procedure was necessary in 4 children in group I.

In the assessment of endoscopic techniques' efficiency in the treatment of nephrolithiasis in children, the patients with congenital abnormalities are of special interest. In our group, three categories of congenital abnormalities were found, namely, primary megaureter, primary vesicoureteral reflux, and ureterocele. Our findings did not indicate that any of these conditions directly affected the results of the endoscopic procedures. In the case of patients with ureterocele, the end results were not affected either as the endoscope overcomes the obstruction and the disintegration of deposits and their extraction from the urinary tract is performed under visual guidance.

A high efficacy of endoscopic nephrolithiasis treatment in patients with congenital abnormalities of the urinary tract was also demonstrated through the results reported by Ugurlu et al. A group of 25 children with congenital kidney abnormalities was treated with an RIRS procedure due to nephrolithiasis. The overall efficacy for this method amounted to 88%, with an SFR of 64% achieved after the first procedure [31].

Based on our observations, it was found that both pneumatic and laser ureterorenoscopic lithotripsies are safe, with a comparable, clinically insignificant percentage of complications in both URSL and RIRS. We found no statistical significance between the two groups in the number of complications. The element in favor of the laser is a significantly shorter operative time in patients treated with this kind of energy. The recorded one case of severe complication in the form of extensive damage to the ureteral wall cannot be considered in the context of the equipment used, but rather with regard to the risks associated with endoscopy in itself.

In the discussed groups of patients, through the careful implementation of ureteroscope to the ureteral orifice, we recorded a small percentage of vesicoureteral reflux, which underwent spontaneous regression. In the postoperative period, there was no development of iatrogenic ureteral stricture wall in either of the patient groups.

The introduction of holmium laser to our center has improved the results of treatment, making the disintegration of stones more efficient, without affecting operations' safety. Based on our observations, the use of ureterorenoscopic lithotripsy with laser energy in the disintegration of deposits located in both the ureter and a pelvicalyceal system may be considered as first-line treatment of stones in children.

## 6. Conclusions

1. The use of holmium lasers for the disintegration of stones reduces the duration of the procedure and increases the effectiveness of treatment, especially in the case of renal stones (RIRS), compared with the treatment using pneumatic lithotripters.
2. The use of an ureterorenoscope in the treatment of urolithiasis in children, for both the location in the ureter and the kidney, is—in the hands of experienced operators—a highly efficient and safe method.

## Figures and Tables

**Table 1:** Urinary tract defects associated with urinary tract stones in both groups.

Urinary tract defects	Group I	Group II
Primary megaureter	2	1
Vesicoureteral reflux	5	6
Ureterocele	3	2
Summary	10 (15.38%)	9 (20.93%)

**Table 2:** Characteristics of the selected parameters of children in group I and group II who underwent URSL procedure.

URSL	Group I	Group II	*p* value
Medium age years (SD)	8.77 (4.1)	7.52 (3.6)	0.3
Sex (%)			
Female	25 (71.43)	10 (28.57)	0.04
Male	21 (75)	7 (25)	0.05
Medium height m (SD)	1.32 (0.39)	1.25 (0.5)	0.3
Medium weight kg (SD)	27.45 (28.55)	26.5 (24.5)	0.3
BMI kg/m2 medium (SD)	17.45 (3.2)	16.58 (5.62)	0.3
SA (SD)	0.744 (1.44)	0.72 (0.9)	0.3
Side (%)			
right	29 (74,36)	10 (25,64)	0.05
left	17 (70,83)	7 (29,17)	0.05

SA: stone surface area (stone length × stone width × 3.14 × 0.25).

Stone length and width estimated on abdominal radiography.

**Table 3:** Characteristics of selected parameters of the children in group I and group II who underwent RIRS procedure.

RIRS	Group I	Group II	*p* value
Medium age years (SD)	8.45 (5.2)	7,67 (4.4)	0.3
Sex (%)			
Female	11 (39.29)	17 (60.71)	0.2
Male	8 (47.06)	9 (52.94)	0.4
Medium height m (SD)	1.45 (0.42)	1.29 (0.45)	0.3
Medium weight kg (SD)	28.75 (29.35)	26.8 (25.74)	0.3
BMI kg/m2 medium (SD)	17.67 (4.2)	16.47 (5.83)	0.3
SA (SD)	1.34 (1.89)	1.19 (1.42)	0.4
Side (%)			
Right	10 (40)	15 (60)	0.3
Left	9 (45)	11 (55)	0.3

**Table 4:** Operative time in both groups.

	Group I	Group II	*p*
Operative time (min.)	56	34	0.04

**Table 5:** Comparison of URSL results in both groups.

URSL	Group I (*n*-46)	Group II (*n*-17)	*p* value
Complete stone disintegration (SFR)	40 (86.96%)	17 (100%)	0.03
Partial disintegration with debris removal	1 (2.17%)	0	0.4
Partial disintegration with debris leave	3 (6.52%)	0	0.3
Failure	2 (4.35%)	0	0.2

**Table 6:** Comparison of RIRS results in both groups.

RIRS	Group I (*n*-19)	Group II (*n*-26)	*p* value
Complete stone disintegration (SFR)	9 (47.37%)	23 (88.46%)	0.03
Partial disintegration with debris removal	6 (31.58%)	1 (3.84%)	0.05
Partial disintegration with debris leave	4 (21.05%)	2 (7.7%)	0.06
Failure	0	0	0.5

**Table 7:** Complications after procedures in both groups.

Complications	Group I	Group II	*p* value
Fever	12 (18.5%)	9 (21%)	0.2
Urinary tract infection	11 (17%)	7 (16,3%)	0.2
Renal colic	13 (20%)	9 (21%)	0.2
Transient hematuria	10 (15%)	5 (11.6%)	0.04
